# Field-Induced Slow Magnetic Relaxation in Mononuclear Cobalt(II) Complexes Decorated by Macrocyclic Pentaaza Ligands

**DOI:** 10.3390/molecules29122810

**Published:** 2024-06-12

**Authors:** Mengmeng Zeng, Zeyu Ruan, Siguo Wu, Mingliang Tong

**Affiliations:** Key Laboratory of Bioinorganic and Synthetic Chemistry of Ministry of Education, School of Chemistry, Sun Yat-Sen University, Guangzhou 510006, China; zengmm6@mail2.sysu.edu.cn (M.Z.); ruanzy@mail2.sysu.edu.cn (Z.R.); tongml@mail.sysu.edu.cn (M.T.)

**Keywords:** single-ion magnet, cobalt(II), trigonal bipyramidal, macrocyclic pentaaza ligand

## Abstract

Two cobalt(II) complexes [CoL_1_](OTf)_2_ (**1**, L_1_ = 6,6′′-di(anilino)-4′-phenyl-2,2′:6′,2′′-terpyridine) and [CoL_2_](OTf)_2_·MeOH (**2**, L_2_ = 6,6′′-di(*N*,*N*-dimethylamino)-4′-phenyl-2,2′:6′,2′′-terpyridine) were synthesized and characterized. Crystal structure analyses showed that the spin carries were coordinated by five N atoms from the neutral pentaaza ligands, forming distorted trigonal bipyramidal coordination environments. Ab initio calculations revealed large easy-axial anisotropy in complexes **1** and **2**. Magnetic measurements suggest that complexes **1** and **2** are field-induced single-molecule magnets, whose relaxations are mainly predominated by Raman and direct processes.

## 1. Introduction

Since the discovery of magnetic bistability and slow magnetic relaxation behavior at a single-molecule level, single-molecule magnets (SMMs) have been regarded as promising candidates for high-density information storage, quantum computing, and spintronic devices [1,2,3,4,5]. For a magnetized SMM with well-defined spin multiplets, the reversal of magnetization needs to overcome an effective energy barrier (*U*_eff_), because of which the magnetic memory effect can persist below blocking temperature (*T*_B_). The *U*_eff_ is related to the total spin *S* and magnetic anisotropy parameter *D*: for integral spin values, *U*_eff_ = |*D*|*S*^2^; for half-integral spin values, *U*_eff_ = |*D*| (*S*^2^ − 1/4) [6]. The early research was focused on increasing *S* in transition metal clusters to obtain a larger *U*_eff_ [7,8,9,10,11]. For instance, the mixed-valent {Mn_19_} aggregate displays a ground state spin of *S* = 83/2 thanks to the ferromagnetic couplings among spin centers. However, the anisotropy in {Mn_19_} is almost cancelled owing to symmetric topology of magnetic centers [11]. A related theoretical study by Neese et al. reveals a negative correlation between *D* and *S*^2^, indicating that pursuing high total *S* values blindly might be futile in improving *U*_eff_ [12]. Instead, mononuclear complexes with a single paramagnetic center, namely single-ion magnets (SIMs), shift the focus from pursuing a large *S* toward tuning the magnetic anisotropy [13,14,15,16].

Cobalt(II), as a Kramers ion with a half-integral spin, is desirable for designing SIMs. On the one hand, quantum tunneling of magnetization (QTM) can be inherently inhibited in Co-SIMs. On the other hand, the magnetic anisotropy in Co-SIMs can be tuned by modulating the coordination environment of the spin carrier. Up to now, Co(II) complexes with different geometries (linear [17,18,19], tetrahedral [20,21,22], square pyramidal [23,24,25], trigonal bipyramidal [26,27,28,29], octahedral [30,31,32,33], triangular prism [34,35,36,37], etc.) had been reported with large magnetic anisotropy and SIM behaviors. The first example of zero-field Co(II)-SIM was the tetrahedral complex [Co(SPh)_4_]^2−^, wherein the *S* = 3/2 ground state is stabilized in *D*_2d_ geometry with the help of soft donor atoms [20]. Up to now, the best-performing Co(II)-SIM was reported with a reversal barrier of 450 cm^−1^, taking advantage of the linear coordination environment which reserves the first-order orbital contribution [19]. Also, the substituent effect of the ligand can be potentially exploited to modulate magnetic anisotropy. Pushing Co(II) out of plane by modifying the pincer ligands enhanced spin–orbit coupling in the [{ArN=CR}_2_(NPh)]Co(NCS)_2_ (R = Me or Ph) system [25].

The ligand field of Co(II) significantly influences the sign and magnitude of *D*. To stabilize a negative *D* (Ising-type or easy-axis anisotropy), it is suggested to place a Co(II) ion with a trigonal bipyramidal geometry. In general, a mix-ligand strategy (the combination of multidentate N-donor and halido/pseudo-halido ligands) is employed when constructing Co(II) complexes with *C*_3_ geometry [38,39,40]. For example, [Co(terpy)Cl_2_] and [Co(terpy)(NCS)_2_] were constructed by employing a terpyridine ligand and exhibited slow magnetization relaxation behaviors through multiple pathways [41]. Nevertheless, enforcing Co(II) with trigonal bipyramidal geometry with one ligand remains a synthetic challenge.

In this study, we aim at developing Co(II)-SIMs with substituted terpyridine ligands and investigate the substituent effect toward structural geometry and magnetic anisotropy. Herein, by using macrocyclic pentaaza ligands, two penta-coordinated Co(II) complexes, [CoL_1_](OTf)_2_ (**1**) and [CoL_2_](OTf)_2_·MeOH (**2**) (L_1_ = 6,6′′-di(anilino)-4′-phenyl-2,2′:6′,2′′-terpyridine, L_2_ = 6,6′′-di(*N*,*N*-dimethylamino)-4′-phenyl-2,2′:6′,2′′-terpyridine), were synthesized with distorted trigonal bipyramidal geometry. Structure investigations and magnetic properties studies were performed on these complexes.

## 2. Results and Discussions

### 2.1. Synthesis and Structure Analysis

Complexes **1** and **2** are synthesized by direct reactions of ligands and metal salts in solvent mixtures of methanol and DCM. Phase purity was confirmed by elemental analysis and powder X-ray diffraction experiments (Figure S1). Thermogravimetric (TG) analysis showed that there is no solvent in the lattice for complex **1**, while a weight loss ratio of 3.3% was consistent with the escape of one methanol molecule for complex **2** (Figure S2).

**Figure 1:**
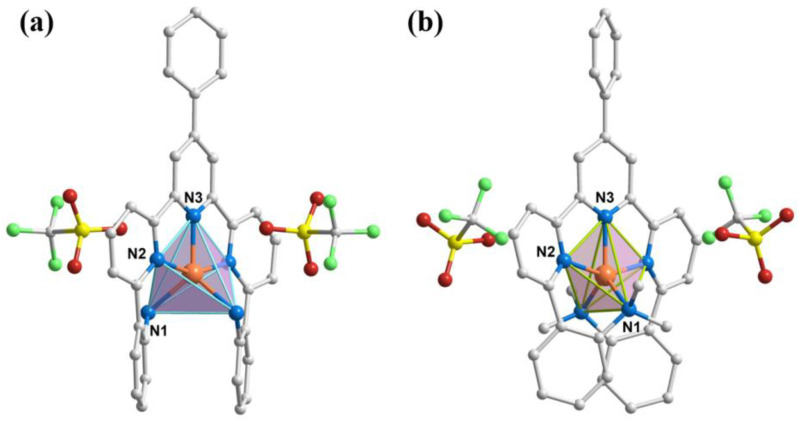
Molecular structures of complexes **1** (**a**) and **2** (**b**). Color code: Co, orange; N, blue; C, grey; O, red; S, yellow; F, green. H atoms and solvent molecules were omitted for clarity.

Single-crystal X-ray diffraction measurements indicate that complexes **1** and **2** both crystallize in the monoclinic space group *C*2/*c*, with four molecules in a unit cell (Table S1). For complex **1**, the asymmetric unit contains half of a Co(II) ion, half of a L_1_ ligand, and one trifluoromethanesulfonate anion (OTf^−^) as the counter ion. For complex **2**, the asymmetric unit contains half of a Co(II) ion, half of a L_2_ ligand, one OTf^−^ anion, and half of a two-fold disordered methanol molecule. Molecular structures of complexes **1** and **2** are shown in Figure 1, and selected bond lengths and angles are listed in Table S2. The Co(II) center ions in the two complexes are coordinated to five N atoms from neutral macrocyclic pentaaza ligands, forming [CoN_5_] coordination environments. To confirm the geometrical configurations of the penta-coordinated complexes, we performed Continuous Shape Measure (CShM) calculations using the Shape 2.1 program [42], and the results are shown in Table S3. The minimum CShM values of complexes **1** and **2** are 5.061 and 3.677, respectively, which corresponds to the trigonal bipyramidal (TBPY) configuration, followed by 6.146 and 5.659, corresponding to the square pyramidal (SPY) configuration. Although the calculated values are comparable, the geometric configurations are more inclined to TBPY ones. In addition, they could be evaluated with an Addison parameter *τ* [43]: with a perfect SPY when *τ* = 0 and a typical TBPY when *τ* = 1. The calculated parameters *τ* are 0.43 for complex **1** and 0.49 for complex **2**. The Co–N bond lengths of complexes **1** and **2** range between 1.992 and 2.103 Å and between 2.019 and 2.129 Å, with average values of 2.074/2.096 Å, indicating a high-spin (HS) state of Co(II) ions.

Despite similar molecular skeletons in complexes **1** and **2**, intermolecular interactions are distinct in these complexes (Figures S4 and S5). For complex **1**, offset intermolecular π⋯π interactions between the pyridine and benzene rings of the ligand L_1_ could be observed among the adjacent molecules with a centroid distance of 3.742 Å. Meanwhile, the OTf^−^ anions form hydrogen bonds with the amino moieties of L_1_ ligands and yield an O⋯H–N distance of 3.007 Å. As for complex **2**, there is no π⋯π stacking effect due to the steric hindrance of two methyl groups in L_2_ ligands as well as the well-separated [CoL_2_]^2+^ by OTf^–^ and methanol molecules. The shortest distances of Co⋯Co for complexes **1** and **2** are 8.918 Å and 10.101 Å, respectively, suggesting no significant magnetic exchange interaction between the metal ions (Figures S4 and S5).

### 2.2. Static Magnetic Properties and Theoretical Calculations

**Figure 2:**
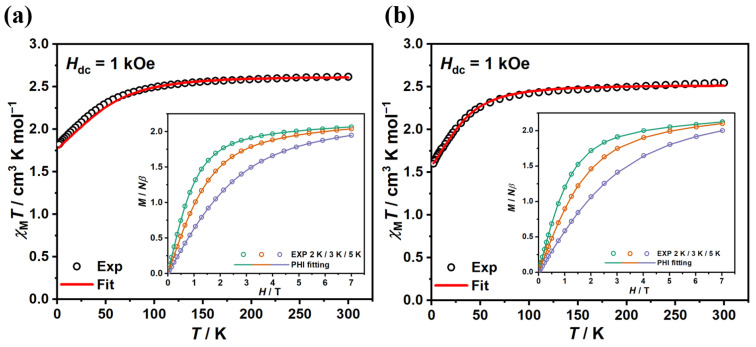
The direct-current (dc) magnetic susceptibilities were collected under a 1 kOe dc field for complexes **1** (**a**) and **2** (**b**), respectively. Inset: Variable-field magnetization data collected from 0 to 7 T in steady fields. Solid lines correspond to the best fit from *PHI*.

Magnetic susceptibility measurements were performed on polycrystalline samples. Temperature-dependent molar magnetic susceptibilities were collected under an external field of 1 kOe in the temperature range 2–300 K (Figure 2), with a similar performance for both complexes. The room temperature *χ*_M_*T* values for complexes **1** and **2** were 2.614 and 2.544 cm^3^ K mol^−1^, respectively, being significantly higher than the theorical spin-only value of 1.875 cm^3^ K mol^−1^ for an isolated Co(II) ion (*S* = 3/2, *g* = 2), indicating the presence of considerable orbital angular momentum contributions. The *χ*_M_*T* value of complex **1** maintained a slow decrease upon cooling to 100 K, after which a sharper drop was observed with a minimum value of 1.821 cm^3^ K mol^−1^ at 2 K. Similarly, the *χ*_M_*T* value for complex **2** decreased more rapidly when the temperature fell below 50 K and reached a minimum value of 1.598 cm^3^ K mol^−1^ at 2 K. The decline with temperature suggests the presence of a weak intermolecular antiferromagnetic interaction and/or zero-field splitting (ZFS) in the complexes. Low-temperature (2/3/5 K) magnetizations data were collected in the range of magnetic field of 0–7 T (Figure 2, inset). The magnetizations increases rapidly below 3 T and hit their maximum of 2.065 and 2.125 *Nβ* at 2 K and 7 T, respectively, being lower than the saturation value of 3 *Nβ* for Co(II) ions. The non-overlapping *M*–*H*/*T* curves for different temperatures demonstrated the presence of magnetic anisotropy (Figure S6).

To further investigate the magnetic anisotropy of the compounds, we analyzed the temperature-dependent susceptibilities and field-dependent magnetizations using *PHI* program [44]. The spin Hamiltonian containing the ZFS and Zeeman effect is shown in Equation (1):

(1) $$\hat{H}=D\left({\hat{S_{z}}}^{2}-S\left(S+1\right)/3\right)+E\left({{\hat{S}}_{x}}^{2}-{{\hat{S}}_{y}}^{2}\right)+\mu_{B}\vec{B}\cdot g\cdot \hat{S}$$

where *D* and *E* represent axial and transverse ZFS parameters, respectively, *S* represents the ground-state spin value, $\vec{B}$ represents the magnetic field vector, and *μ*_B_ represents the Bohr magneton constant. As listed in Table 1, the best-fit result indicates a large easy-axis anisotropy in complex **1** with *D* = −62.7 cm^−1^, *E*/|*D*| = 0.149, *g*_x_ = *g*_y_ = 2.26, *g*_z_ = 2.52. For complex **2**, two sets of parameters can be obtained depending on the sign of the *D* value. With an easy-axis anisotropy, best fitting yields *D* = −34.9 cm^−1^, *E*/|*D*| = 0.309, *g*_x_ = *g*_y_ = 2.28, *g*_z_ = 2.38; with an easy-plane anisotropy, best fitting yields *D* = 36.8 cm^−1^, *E*/|*D*| = 0.33, and *g*_x_ = *g*_y_ = 2.40, *g*_z_ = 2.11. As the rhombicity was non-negligible and the *E*/|*D*| ratios approached 1/3 in both cases, the sign of *D* parameter could not be determined unambiguously [45,46].

To further verify the magnetic anisotropy of complexes **1** and **2**, ab initio calculations were performed in OpenMolcas 24.02 software using the CASSCF/RASSI method. The variations in the main values of *g* tensors between the ground and first excited doublets with pseudo-spin *S* = 1/2 indicate the variations of the magnetic anisotropy in each pair of doublets in Co(II) (Table S4). Regarding the lowing-lying doublets as a whole, the calculated ZFS parameters *D* and *E* as well as ***g*** factors for the ground states (*S* = 3/2) of Co(II) are listed in Table 1 (More details in ESI). The signs and magnitudes of the calculated parameters *D* are comparable to the experimental fits for both complexes, supporting large easy-axial anisotropy. The predicted dc data (*χ*_M_*T*-*T* and *M*-*H*) exhibit similar tendencies with large values compared to the experimental ones and can be basically overlapped after scaling down by various degrees (Figures S7 and S8). However, at extremely low temperatures, the simulated and calculated *χ*_M_*T*-*T* curves slightly deviate from the static magnetic data (Figure 2a and Figure S7a). This might be related to intermolecular magnetic interaction since only single-ion behaviors were considered in *PHI* simulations and ab initio calculations. The calculated energy gaps between the ground and the first excited doublets of Co(II) are 169.9 and 116.5 cm^−1^ for complexes **1** and **2**, respectively (Figure S9 and Table S4).

### 2.3. Dynamic Magnetic Properties

Alternative-current (ac) magnetic susceptibility measurements were performed on complexes **1** and **2**. Under a zero-applied field, no out-of-phase (*χ*″_M_) signal was observed in either of the two complexes (Figure S10), which could be attributed to the presence of the QTM effect. To suppress the QTM effect, field-dependent ac magnetic susceptibility measurements were collected at 2 K under 0–5 kOe (Figures S11 and S12). Below a 1.5 kOe dc field, the *χ*″_M_ signals exhibited one set of frequency-dependent ac susceptibilities, and the *χ*″_M_ peaks moved to lower frequency with an increase in the dc field. At higher dc fields, another set of *χ*″_M_ signals appeared at a low frequency owing to the dipolar interactions.

**Figure 3:**
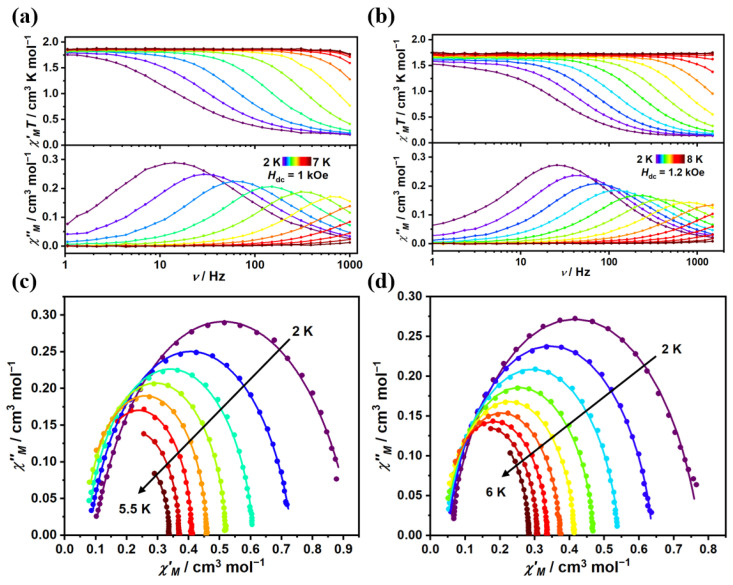
Frequency dependence of the in-phase (*χ*′_M_*T*) and out-of-phase (*χ*″_M_) for complex **1** (**a**) at 1 kOe dc field and for complex **2** (**b**) at 1.2 kOe dc field. The solid lines are guides for the eyes. Cole-Cole plots for complexes **1** (**c**) and **2** (**d**). The solid lines are the best fit to Debye’s law.

For complex **1**, the ac susceptibilities were measured with a 1 kOe applied dc field. As shown in Figure 3a, the clear frequency dependence in both the in-phase *χ*′_M_*T* product and out-of-phase (*χ*″_M_) product was exhibited in a temperature range of 2–7 K. Upon cooling, the maxima of *χ*″_M_ signals moved toward a lower frequency, featuring typical field-induced SMM behavior. For complex **2**, ac susceptibilities were measured at a range of 2–8 K under a 1.2 kOe dc field (Figure 3b). The maximum peak temperature was 5.5 K for complex **2**, being slightly higher than that in complex **1** (4.5 K, Figure S13). The ac data were fitted by the generalized Debye model, and the relaxation time (*τ*) and related parameters are listed in Tables S7 and S8. As depicted in Figure 3c,d, semicircle-like Cole-Cole plots for complexes **1** and **2** revealed that only one relaxation process occurred at the corresponding applied field. The α parameters varying in the ranges of 0–0.23 and 0.03–0.18 indicated a narrow distribution of the relaxation time. 

**Figure 4:**
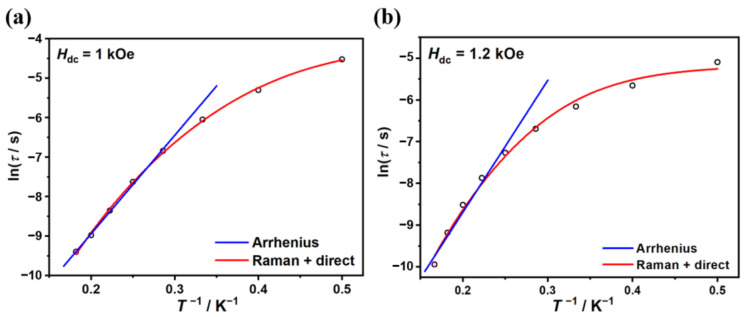
Temperature dependence of the relaxation time *τ* under external dc field for complexes **1** (**a**) and **2** (**b**). The blue lines represent the Arrhenius fit. The red lines represent the best fit of direct and Raman processes.

As shown in Figure 4, the ln(*τ*)*-T^−^*^1^ plots in the high-temperature region can be fitted by the Arrhenius law *τ* = *τ*_0_ exp(*U*_eff_/*k*_B_*T*), providing *U*_eff_ = 25.0(6) K (*τ*_0_ = 9(1) × 10*^−^*^7^ s) for complex **1** and *U*_eff_ = 31(3) K (*τ*_0_ = 3(2) × 10*^−^*^7^ s) for complex **2**. Since the phenomenological reversal barriers are much lower than the values of the first excited doublets from ab initio calculations and the ln(*τ*)*-T^−^*^1^ plots deviate from linearity at lower temperatures, the relaxation dynamics are possibly dominated by under-barrier mechanisms. Taking direct and Raman processes into account (*τ^−^*^1^ = *AT* + *CT^n^*), the best-fit parameters are *n* = 5.8(1), *C* = 0.6(1) s*^−^*^1^ K*^−^*^5.8^, and *A* = 13(2) s*^−^*^1^ K*^−^*^1^ for complex **1** and *n* = 6.1(4), *C* = 0.3(2) s*^−^*^1^ K*^−^*^6.1^, and *A* = 40(7) s*^−^*^1^ K*^−^*^1^ for complex **2**. For Kramers ions, the expected Raman exponent value is *n* = 9. However, in different criteria of real molecules, *n* ranging 1~6 is also reasonable considering optical or acoustic phonons.

## 3. Materials and Methods

### 3.1. Physical Measurements

All reactions and manipulations described below were performed under aerobic conditions. Ligands L_1_ and L_2_ were prepared according to the reported methods [47]. Metal salts and other reagents were commercially available and used as received without further purification. C, H, N, and S elemental analyses were carried out with an Elementar Vario-EL CHNS elemental analyzer (Elementar, Langenselbold, Germany). FT-IR spectra were recorded from KBr pellets in a range of 4000–400 cm^−1^ on a PerkinElmer Frontier FT-IR Spectrometer (PerkinElmer, Waltham, MA, USA). Thermogravimetric analysis (TGA) was carried out on a NETZSCH TG209F1 thermogravimetric analyzer (NETZSCH, Selb, Germany) in a N_2_ atmosphere with a temperature range of room temperature to 800 °C. Magnetic susceptibility measurements were all collected using a Quantum Design MPMS3 SQUID VSM magnetometer (Quantum Design, San Diego, CA, USA). Polycrystalline samples were embedded in Vaseline to prevent torque.

### 3.2. Synthetic Procedure

[CoL_1_](OTf)_2_ (**1**): igand L_1_ (0.04 mmol, 19.7 mg) and metal salt Co(OTf)_2_ (0.04 mmol, 14.3 mg) were dissolved in 5 mL of a mixed solution comprising dichloromethane (DCM) and methanol (*v*:*v* = 2:3). The mixture was stirred at room temperature for 60 min to obtain a yellow solution and was then filtered. The filtrate was slowly diffused with diethyl ether over a period of time to obtain orange-red rhombic crystals suitable for X-ray single-crystal diffraction in a yield of ca. 60% (based on Co). Anal. calcd(%) for C_35_H_25_N_5_O_6_F_6_S_2_Co: C, 49.53; H, 2.97; N, 8.25; S, 7.56. Found(%): C, 49.50; H, 2.86; N, 8.07; S, 7.45. IR spectra (KBr, cm^−1^): 1615 (m), 1601 (m), 1565 (m), 1506 (w), 1475 (m), 1460 (m), 1440 (m), 1385 (m), 1293 (s), 1242 (vs), 1183 (m), 1163 (s), 1140 (s), 1101 (w), 1085 (w), 1061 (w), 1030 (vs), 951 (w), 897 (w), 869 (w), 897 (w), 869 (w), 869 (w), 825 (m), 766 (s), 753 (m), 687 (m), 659 (m), 640 (s), 572 (m), 515 (m), 492 (w).

[CoL_2_](OTf)_2_·MeOH (**2**): The synthesis procedure was the same as complex **1**, except that ligand L_2_ (0.04 mmol, 19.7 mg) was used instead of L_1_. The yellow solution obtained was diffused by ether to form brownish-yellow block crystals in a yield of ca. 60% (based on Co). Anal. calcd(%) for C_40_H_37_N_5_O_7_F_6_S_2_Co: C, 51.28; H, 3.98; N, 7.48; S, 6.84; Found(%): C, 50.94; H, 4.05; N, 7.43; S, 6.87. IR spectra (KBr, cm^−1^): 1610 (m), 1599 (m), 1566 (m), 1549 (w), 1469 (m), 1455 (w), 1437 (m), 1385 (w), 1270 (vs), 1225 (s), 1196 (vw), 1152 (s), 1058 (vw), 1031 (s), 956 (vw), 906 (w), 889 (vw), 831 (w), 786 (m), 775 (m), 763 (m), 739 (w), 705 (w), 657 (w), 639 (s), 574 (w), 535 (w), 517 (m), 458 (w).

### 3.3. X-ray Crystallography

X-ray powder diffraction intensities for polycrystalline samples were measured at room temperature on a RIGAKU D-MAX 2200 VPC diffractometer (RIGAKU, Tokyo, Japan). Single-crystal X-ray measurements were performed with a BRUKER D8 VENTURE PHOTON III diffractometer (Ga-*K*_α_ radiation, *λ* = 1.34138 Å, BRUKER, Mannheim, Germany) for complex **1** and a SuperNova Dual Cu at home/near AtlasS22 diffractometer (Mo-*K*_α_ radiation, *λ* = 0.71073 Å, RIGAKU, Japan) for complex **2** at 150 K, respectively. The single-crystal structures were solved using intrinsic phasing methods (SHELXT) and were refined by SHELXL in Olex2 1.5 program [48,49,50]. The crystal data for all complexes were deposited in the Cambridge Structural Database (CCDC 2343827-2343828 for complexes **1** and **2**).

### 3.4. Computational Details

Ab initio calculations were performed on the crystal structures by using the CASSCF/RASSI method on OpenMolcas version 24.02 [51,52,53]. The cobalt fragment was calculated by employing the ANO-RCC-VTZP basis set for the Co atom, ANO-RCC-VDZP for S, O, and N atoms, as well as ANO-RCC-VDZ for the rest of the atoms. The Cholesky decomposition threshold was set to 1.0·10^−8^ in all calculations. The active space of the CASSCF calculation included 7 electrons in 5 orbitals (3d orbitals of Co^2+^ ion) for the Co calculation. Spin–orbit coupling was considered within the SO-RASSI program. In case of a Co center, all the 10 quartet and 40 doublet states obtained from the CASSCF were mixed by spin–orbit coupling. Based on the resulting spin–orbital multiplets, the SINGLE_ANISO program computed local magnetic properties (*g*-tensors, local magnetic susceptibility, etc.) of the Co(II) ions.

## 4. Conclusions

In summary, two mononuclear cobalt(II) complexes, **1** and **2**, were synthesized successfully using macrocyclic pentaaza ligands. The geometric configurations of the Co(II) ions are restricted to a highly distorted trigonal bipyramid by means of the two N-containing substituents of the terpyridine ligands. Magnetic measurements and ab initio calculations indicated strong easy-axis anisotropy in both complexes with field-induced single-molecule magnets behaviors, and the slow magnetic relaxation processes were dominated by Raman and direct mechanisms owing to non-negligible rhombicity.

## Figures and Tables

**Table 1:** Parameters obtained from *PHI* fitting and theoretical calculations for complexes **1** and **2**.

	1	2	
*PHI* simulations			
*D*/cm^−1^	−62.7 (4)	−34.9 (1)	36.8 (3)
*E*/cm^−1^	9.4 (1)	10.8 (1)	12.4 (1)
*E*/|*D*|	0.149	0.309	0.33
*g*_x_, *g*_y_	2.26	2.28	2.40
*g*_z_	2.52	2.38	2.11
Theoretical calculations			
*D*/cm^−1^	−81.5	−50.9	
*E*/cm^−1^	13.8	16.4	
*E*/|*D*|	0.169	0.322	
*g*_x_, *g*_y_, *g*_z_	2.045, 2.196, 2.976	2.006, 2.308, 2.689	

## Data Availability

Data are contained within the article and Supplementary Materials.

## Supplementary Materials

The following supporting information can be downloaded at: https://www.mdpi.com/article/10.3390/molecules29122810/s1, Figure S1. PXRD patterns of complexes **1** (up) and **2** (down) compared with the simulated patterns from the single-crystal structures. Figure S2. TG analysis of complexes **1** (a) and **2** (b) under N_2_ atmosphere (10 K min^−1^). The theoretical weight loss percentage of one MeOH is 3.4% for complex **2**. Figure S3. Infrared spectra for complexes **1** and **2**. Figure S4. Packing diagrams of complex **1** viewed from a-axis(a) and b-axis(b). Blue dashed lines: hydrogen bonds; pink dashed lines: π⋯π interactions; orange dashed lines: Co∙∙∙Co distances. Figure S5. Packing diagrams of complex **2** viewed from a-axis(a) and b-axis(b). Blue dashed lines: hydrogen bonds; orange dashed lines: Co∙∙∙Co distances. Figure S6. *M* vs. *H*/*T* curves of complexes **1** (a) and **2** (b). The solid lines are the best-fit results using *PHI* program. Figrue S7. Magnetic susceptibilities performed on the powder samples of **1** (a) and **2** (b). The solid lines indicate the scaled down results from ab initio calculations. Figrue S8. *M*-*H* curves for **1** (a) and **2** (b). The solid lines represent the scaled down results from ab initio calculations. Figure S9. Calculated energy levels and relaxation mechanisms for complexes **1** (a) and **2** (b). The red dashed lines correspond to QTM; the blue and green lines represent the spin-phonon transitions. The numbers next to arrows connecting two states display the average transition magnetic moment matrix element between the respective states. Figure S10. Temperature dependence of the in-phase (*χ*′_M_) and out-of-phase (*χ*″_M_) components of the ac magnetic susceptibility for complexes **1** (a) and **2** (b) under zero field. Figure S11. (a) Frequency dependence of the in-phase (*χ*′_M_) and out-of-phase (*χ*″_M_) components at 2 K under different applied fields (0–5000 Oe) for complex **1**. The solid lines are guides for the eyes. (b) The field-dependent relaxation times at 2 K for complex **1**. Figure S12. (a) Frequency dependence of the in-phase (*χ*′_M_) and out-of-phase (*χ*″_M_) components at 2 K under different applied fields (0–5000 Oe) for complex **2**. The solid lines are guides for the eyes. (b) The field-dependent relaxation times at 2 K for complex **2**. Figure S13. Temperature dependence of the in-phase (*χ*′_M_*T*) and out-of-phase (*χ*″_M_) for complex **1** (a) at 1 kOe dc field and for complex **2** (b) at 1200 Oe dc field. The solid lines are guides for the eyes. Table S1. Crystallographic data of complexes 1 and **2**. Table S2. Selected bonds lengths [Å] and angles [°] for complexes **1** and **2**. Table S3. Continuous shape measures calculations (CShM) for transition metal ions in complexes **1** and **2**. Table S4. Calculated energy levels (cm^−1^), main value of ***g*** tensors (*g*_x_, *g*_y_, *g*_z_) of the ground, and first excited doublets of the Co(II) ions in complexes **1** and **2** (effective ***S*** = 1/2). Table S5. Calculated main value of ***g*** tensors (*g*_x_, *g*_y_, *g*_z_) and ***D*** tensors (*D*_x_, *D*_y_, *D*_z_) of the ground states of the Co(II) ions in complexes **1** and **2** (effective ***S*** = 3/2). Table S6. Calculated low-lying spin–orbit energies (cm^−1^) of the Co(II) ions in complexes **1** and **2**. Table S7. Parameters from the fitting of Cole–Cole plots by the generalized Debye model at 1 kOe dc field for complex **1**. Table S8. Parameters from the fitting of Cole–Cole plots by the generalized Debye model at 1.2 kOe dc field for complex **2**. Reference [54] is cited in the Supplementary Materials.
