# Supplementary material for: Field-Induced Slow Magnetic Relaxation in Mononuclear Cobalt(II) Complexes Decorated by Macrocyclic Pentaaza Ligands
Source: Molecules. 2024 Jun 12;29(12):2810. doi: 10.3390/molecules29122810 (PMC11206533; doi:10.3390/molecules29122810)
Supplement: Supplementary file 1 [file molecules-29-02810-s001.zip › molecules-3026003-supplementary.pdf]

Supporting Information

Field-induced slow magnetic relaxation in mononuclear cobalt(II) complexes decorated by macrocyclic pentaaza ligands

Mengmeng Zeng, Zeyu Ruan, Siguo Wu*, Mingliang Tong

Key Laboratory of Bioinorganic and Synthetic Chemistry of Ministry of Education, School of Chemistry, Sun Yat-Sen University, Guangzhou 510006, China

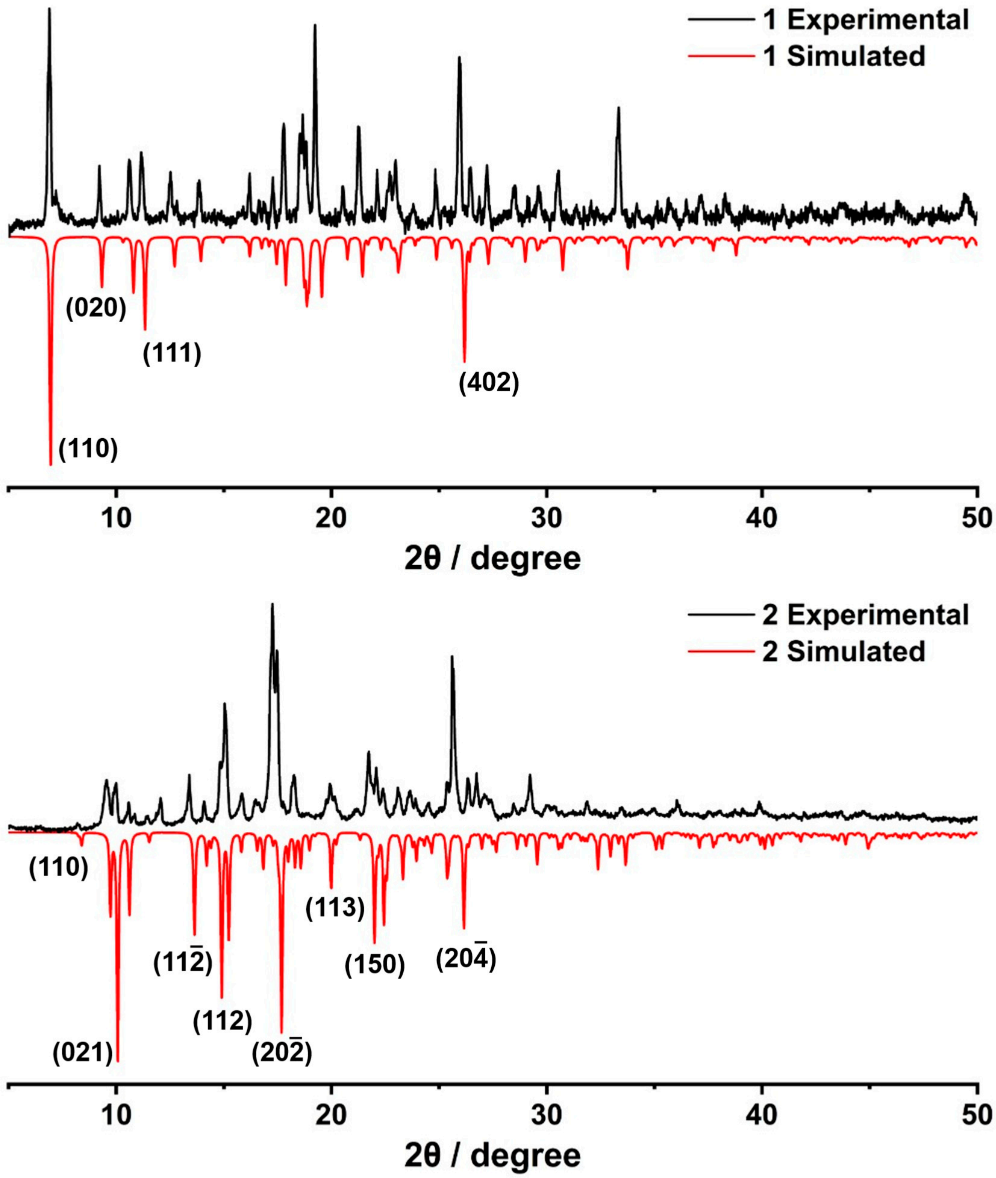

Figure S1. PXRD patterns of **1** (up) and **2** (down) compared with the simulated patterns from the single-crystal structures.

The positions of main peaks in experimental data are basically consisted with the theoretical ones. And the slight deviation is due to the different testing temperature for SC-XRD (150 K) and PXRD (room-temperature) measurements. The change in intensity of some peaks in PXRD patterns can be attributed to the preferred orientation effect caused by uneven grinding.

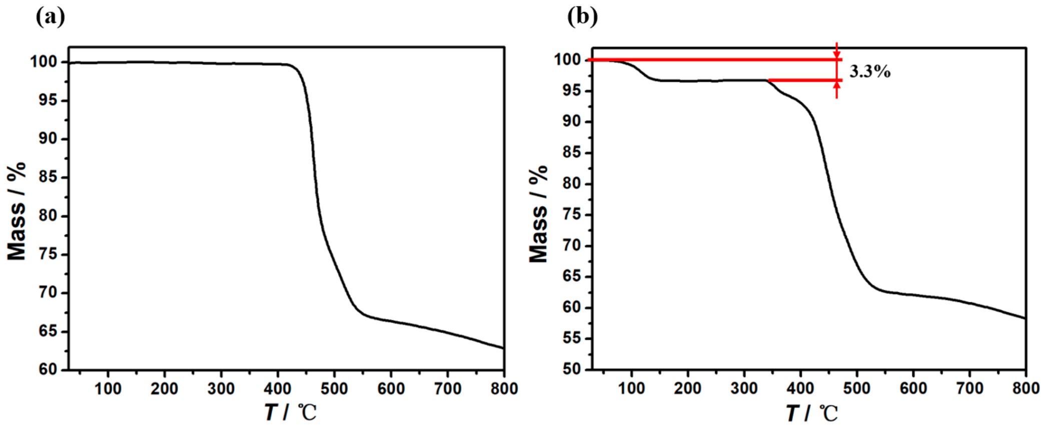

Figure S2. TG analysis of **1** (a) and **2** (b) under N_2 atmosphere (10 K min^{-1}). The theoretical weight loss percentage of one MeOH is 3.4% for **2**.

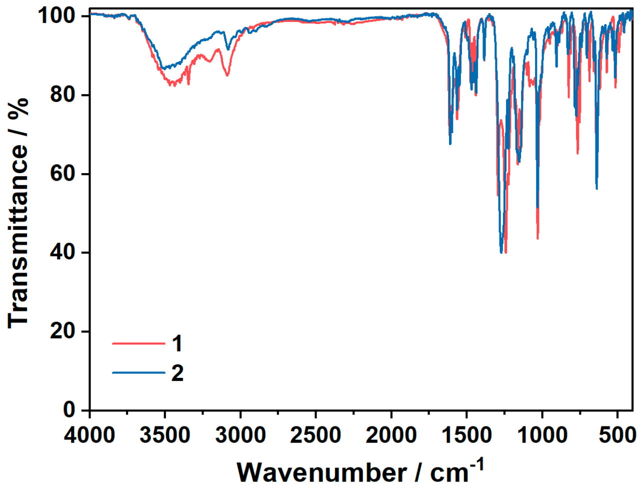

Figure S3. Infrared spectra for **1** and **2**.

Table S1. Crystallographic data of **1** and **2**.

Complex	1	2
**Chemical formula**	$C_{35}H_{25}CoF_6N_5O_6S_2$	$C_{40}H_{37}CoF_6N_5O_7S_2$
**Formula weight**	848.65	936.79
**Temperature/K**	150	149.98(10)
**Crystal system**	Monoclinic	Monoclinic
**Space group**	*C*2/*c*	*C*2/*c*
a **/ Å**	17.1666(7)	12.1425(9)
b **/ Å**	18.9254(7)	21.3848(13)
c **/ Å**	10.2867(4)	15.4036(12)
α **/ °**	90	90
β **/ °**	93.8120(10)	95.967(7)
γ **/ °**	90	90
V **/ Å^3**	3334.6(2)	3978.1(5)
Z	4	4
ρ_{calc} **/ g·cm^{-3}**	1.69	1.564
μ **/ mm^{-1}**	4.118	0.623
F**(000)**	1724	1924
**Crystal size/mm^3**	0.1 × 0.06 × 0.03	0.3 × 0.1 × 0.03
**Radiation / Å**	Ga$K\alpha$ (λ = 1.34138)	Mo$K\alpha$ (λ = 0.71073)
**Reflections collected**	28939	11024
**Independent reflections**	3652 [R_{int} = 0.0435, R_{sigma} = 0.0299]	4804 [R_{int} = 0.0546, R_{sigma} = 0.0852]
**Goodness-of-fit on F^2**	1.065	1.025
**Final R indexes [I>=2σ (I)]**	R_1 = 0.0328, wR_2 = 0.0955	R_1 = 0.0674, wR_2 = 0.1361
**Final R indexes [all data]**	R_1 = 0.0362, wR_2 = 0.1089	R_1 = 0.1012, wR_2 = 0.1552
**Largest diff. peak/hole/e Å^{-3}**	0.61/-0.74	0.79/-0.62
**CCDC No.**	2343828	2343827

$^aR_1 = \Sigma||F_o| - |F_c||/\Sigma|F_o|$; $^bwR_2 = [\Sigma w(F_o^2 - F_c^2)^2 / \Sigma w(F_o^2)^2]^{1/2}$.

Table S2. Selected bonds lengths [Å] and angles [°] for **1** and **2**.

**1**		**2**	
**Bond**	**Length / Å**	**Bond**	**Length / Å**
Co1−N1	2.0854(13)	Co1−N1	2.129(3)
Co1−N1^1	2.0854(13)	Co1−N1^2	2.129(3)
Co1−N2	2.1030(12)	Co1−N2	2.101(3)
Co1−N2^1	2.1030(12)	Co1−N2^2	2.101(3)
Co1−N3	1.9916(17)	Co1−N3	2.019(4)
**Bond**	**Angle / °**	**Bond**	**Angle / °**
N1−Co1− N1^1	99.29(7)	N1−Co1− N1^2	110.33(15)
N1−Co1−N2	82.59(5)	N1−Co1−N2	83.30(10)
N1−Co1−N2^1	82.59(5)	N1−Co1−N2^2	111.75(10)
N1−Co1−N3	130.36(4)	N1−Co1−N3	124.84(8)
N1^1−Co1−N2	113.32(5)	N1^2−Co1−N2	111.74(10)
N1^1−Co1−N2^1	82.59(5)	N1^2−Co1−N2^2	83.30(10)
N1^1−Co1−N3	130.36(4)	N1^2−Co1−N3	124.84(8)
N2−Co1−N2^1	156.21(7)	N2−Co1−N2^2	154.33(15)
N2−Co1−N3	78.11(3)	N2−Co1−N3	77.16(7)
N2^1−Co1−N3	78.11(3)	N2^2−Co1−N3	77.16(7)

^{1}: 1-X, +Y, 3/2-Z; ^{2}: 1-X, +Y, 1/2-Z.

Table S3. Continuous shape measures calculations (CShM) for transition metal ions in **1** and **2**.

**Structure [ML_5]**	**PP-5**	**vOC-5**	**TBPY-5**	**SPY-5**	**JTBPY-5**
**1**	18.891	7.993	5.061	6.146	7.591
**2**	22.761	8.110	3.677	5.659	6.776

PP-5(D_{5h}) = Pentagon; vOC-5(C_{4v}) = Vacant octahedron; TBPY-5(D_{3h}): Trigonal bipyramid; SPY-5(C_{4v}) = Spherical square pyramid; JTBPY-5(D_{3h}) = Johnson trigonal bipyramid J12.

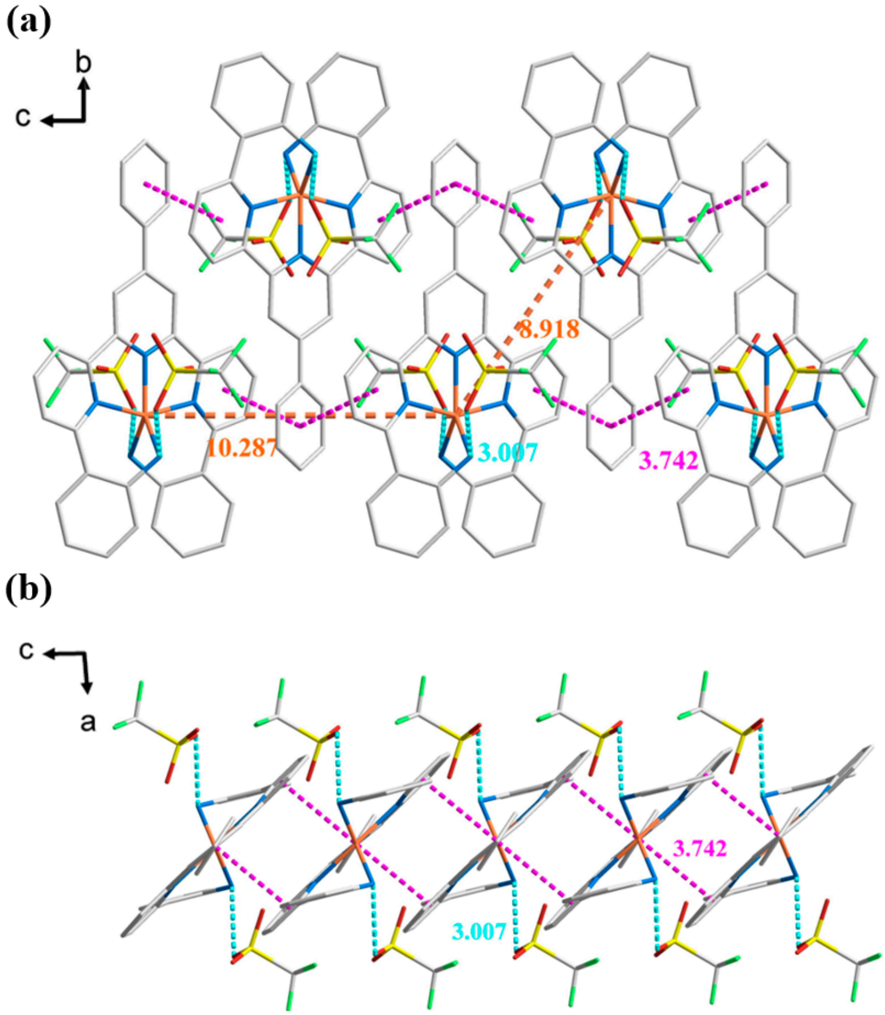

Figure S4. Packing diagrams of **1** viewing from *a*-axis(a) and *b*-axis(b). Blue dashed lines: hydrogen bonds; pink dashed lines: π···π interactions; orange dashed lines: Co···Co distances.

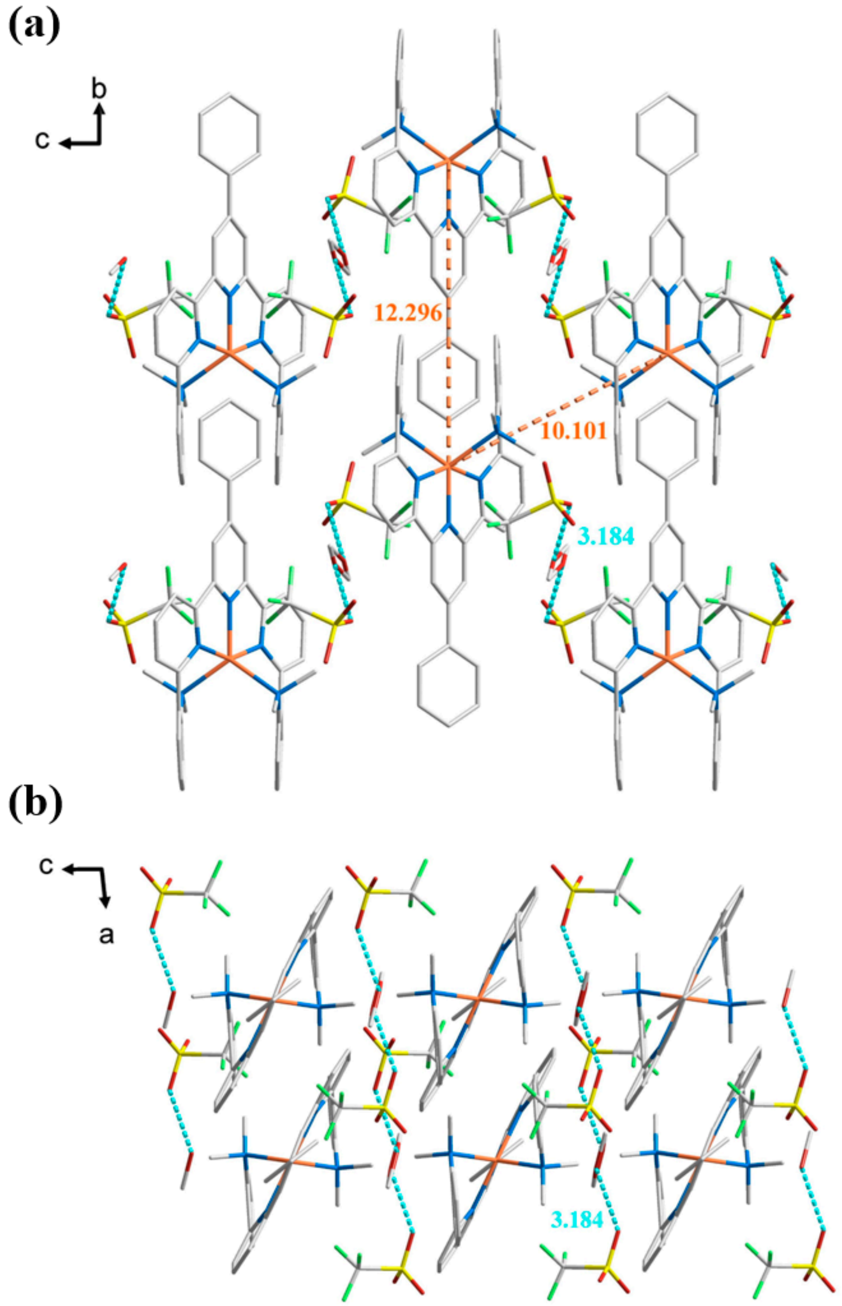

Figure S5. Packing diagrams of **2** viewing from *a*-axis(a) and *b*-axis(b). Blue dashed lines: hydrogen bonds; orange dashed lines: Co···Co distances.

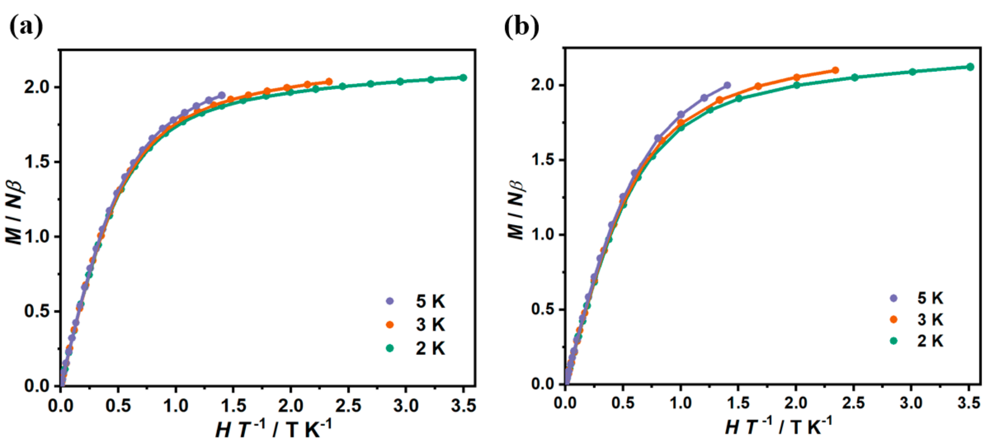

Figure S6. *M vs H*/*T* curves of **1** (a) and **2** (b). The solid lines are the best-fit results using *PHI* program.

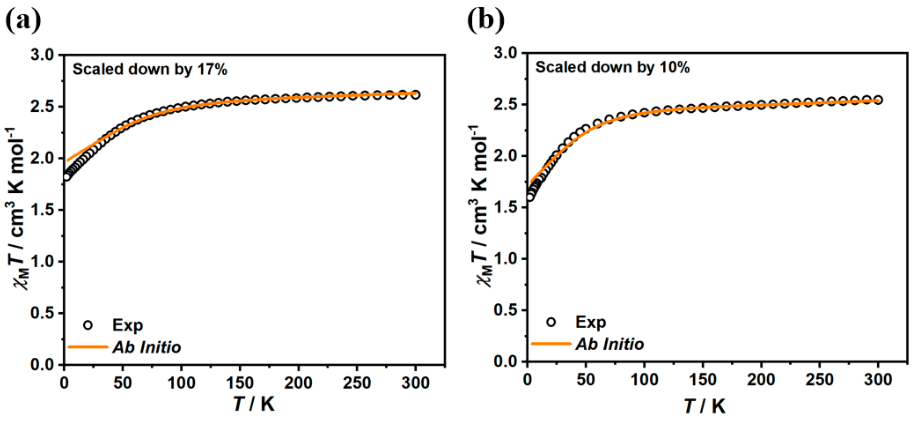

Figrue S7. Magnetic susceptibilities performed on the powder samples of **1** (a) and **2** (b). The solid lines indicate the scaled down results from *ab initio* calculations.

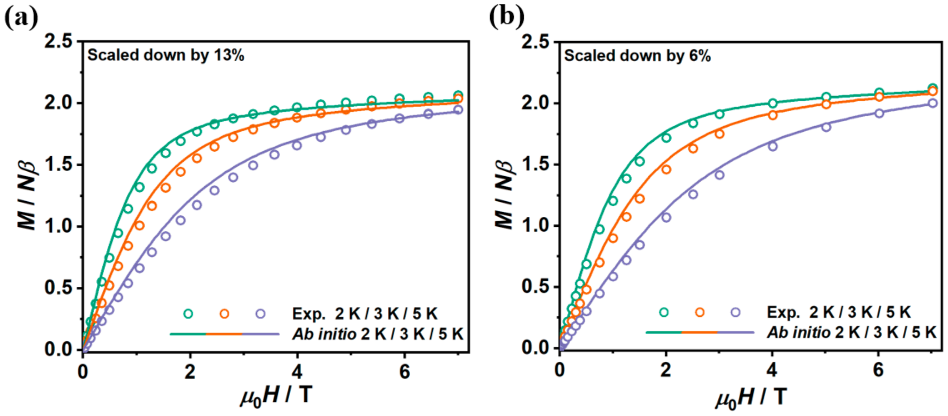

Figrue S8. *M*-*H* curves for **1** (a) and **2** (b). The solid lines represent the scaled down results from *ab initio* calculations.

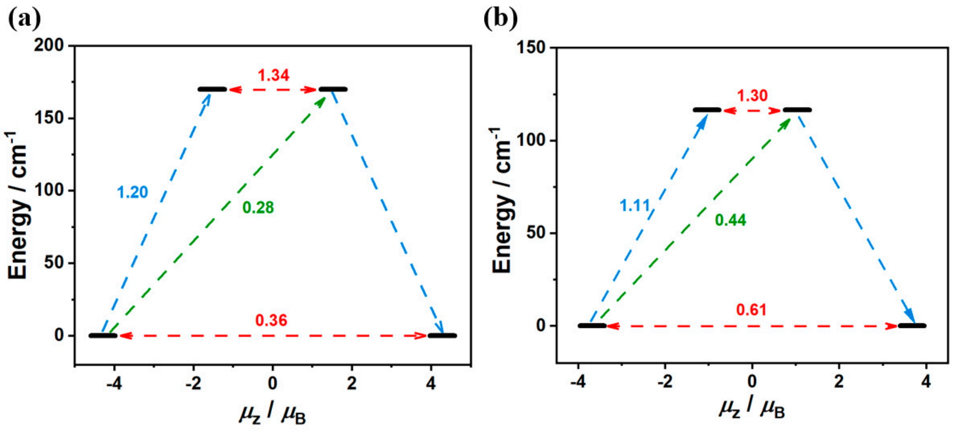

Figure S9. Calculated energy levels and relaxation mechanisms for **1**(a) and **2**(b). The red dashed lines correspond to QTM; the blue and green lines represent the spin-phonon transitions. The numbers next to arrows connecting two states display the average transition magnetic moment matrix element between the respective states.

Table S4. Calculated energy levels (cm^{-1}), main value of ***g*** tensors (g_x, g_y, g_z) of the ground and first excited doublets of the Co(II) ions in complex **1** and **2** (effective $\boldsymbol{S}$ = 1/2).

	**1**		**2**	
**KDs**	***E*** **/ cm^{-1}**	***g***	***E*** **/ cm^{-1}**	***g***
1	0.0	1.004<br>1.171<br>8.570	0.0	1.445<br>2.232<br>7.356
2	169.9	3.051<br>3.138<br>4.898	116.5	2.075<br>2.371<br>5.438

Table S5. Calculated main value of ***g*** tensors (g_x, g_y, g_z) and ***D*** tensors (D_x, D_y, D_z) of the ground states of the Co(II) ions in complex **1** and **2** (effective $\boldsymbol{S}$ = 3/2).

**1**		**2**	
g	***D***	***g***	***D***
2.045	40.984	2.006	33.362
2.196	13.342	2.308	0.538
2.976	-54.326	2.689	-33.900

Thus, we can calculate anisotropy parameters *D* and *E* by[54]:

$$D = \frac{3}{2} \times D_z;$$

$$E = (D_x - D_y)/2$$

Table S6. Calculated low-lying spin-orbit energies (cm^{-1}) of the Co(II) ions in complex **1** and **2**.

low-lying spin-orbit energies	**1**	**2**
	E / cm^{-1}	E / cm^{-1}
1	0	0
2	0	0
3	169.863884	116.5131984
4	169.863884	116.5131984
5	796.8822379	1038.021346
6	796.8822379	1038.021346
7	1089.487955	1477.817548
8	1089.487955	1477.817548
9	2020.48449	2020.310082
10	2020.48449	2020.310082
11	2136.055071	2346.717453
12	2136.055071	2346.717453
13	4353.523645	3676.11096
14	4353.523645	3676.11096
15	4420.325953	3701.614467
16	4420.325953	3701.614467
17	6041.084446	5005.900351
18	6041.084446	5005.900351
19	6085.936983	5080.380575
20	6085.936983	5080.380575

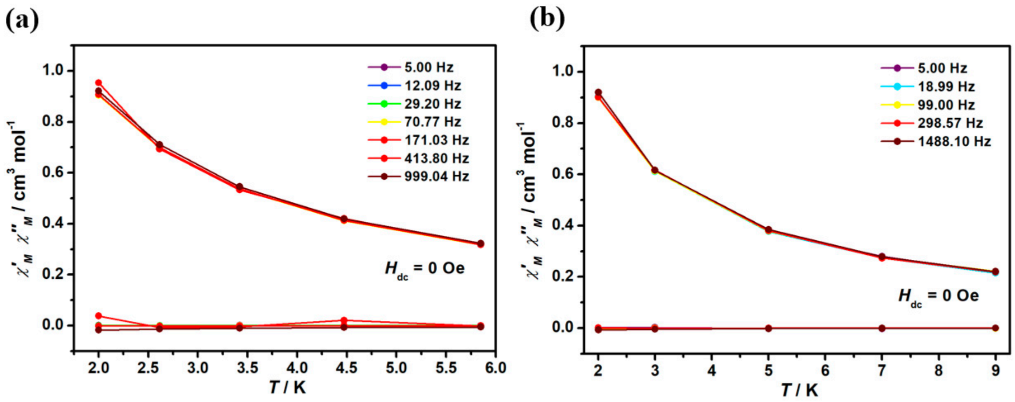

Figure S10. Temperature dependence of the in-phase (χ'_M) and out-of-phase (χ''_M) components of the ac magnetic susceptibility for **1**(a) and **2**(b) under zero field.

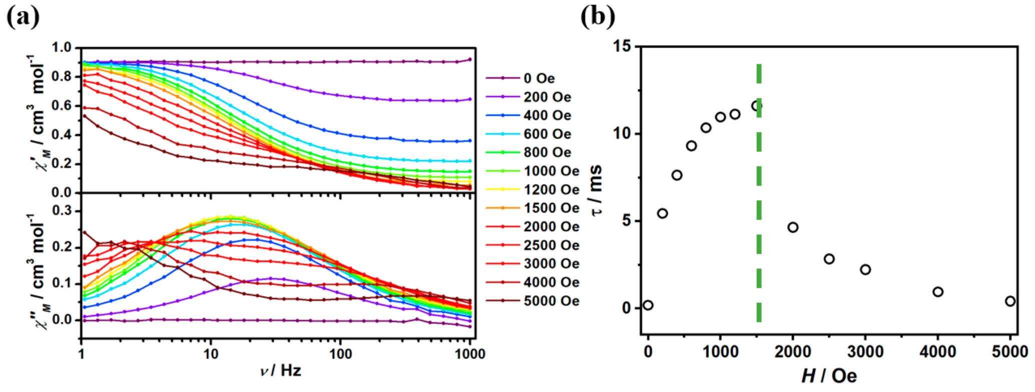

Figure S11. (a) Frequency dependence of the in-phase (χ'_M) and out-of-phase (χ''_M) components at 2 K under different applied fields (0−5000 Oe) for **1**. The solid lines are guides for the eyes. (b) The field-dependent relaxation times at 2 K for **1**.

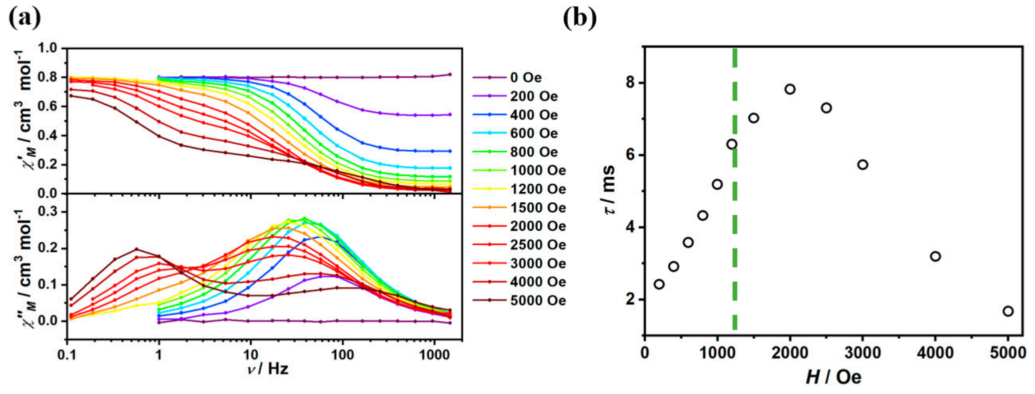

Figure S12. (a) Frequency dependence of the in-phase (χ'_M) and out-of-phase (χ''_M) components at 2 K under different applied fields (0−5000 Oe) for **2**. The solid lines are guides for the eyes. (b) The field-dependent relaxation times at 2 K for **2**.

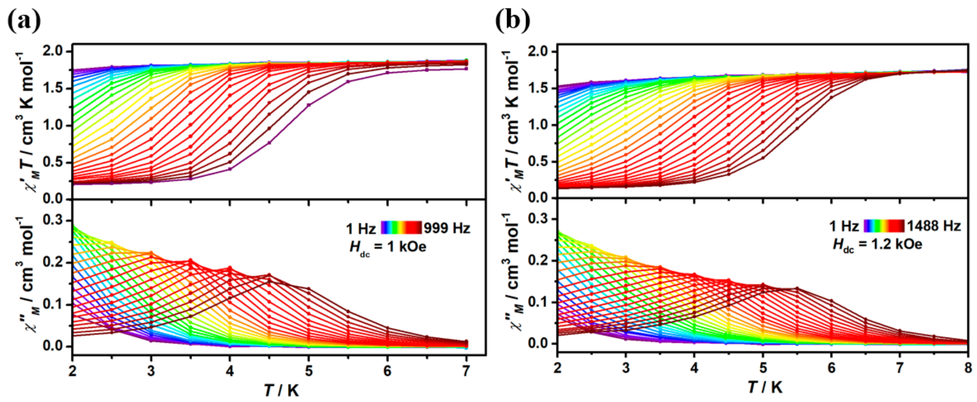

Figure S13. Temperature dependence of the in-phase ($\chi'_{\mathrm{M}}T$) and out-of-phase (χ''_{M}) for **1**(a) at 1 kOe dc field and for **2**(b) at 1200 Oe dc field. The solid lines are guides for the eyes.

Table S7. Parameters from the fitting of Cole-Cole plots by the generalized Debye model at 1 kOe dc field for **1**.

T **/ K**	***τ*** **/ s**	***α***	χ_S **/ cm³ mol⁻¹**	χ_T **/ cm³ mol⁻¹**
2	1.086×10^{-2}	0.22555	0.93075	0.09516
2.5	4.98×10^{-3}	0.16804	0.73356	0.08002
3	2.37×10^{-3}	0.10912	0.6098	0.0723
3.5	1.07×10^{-3}	0.06085	0.5213	0.0653
4	4.86×10^{-4}	0.02471	0.45813	0.06347
4.5	2.36×10^{-4}	0	0.40946	0.0693
5	1.26×10^{-4}	0	0.36895	0.08697
5.5	8.30×10^{-5}	0	0.33661	0.13467

Table S8. Parameters from the fitting of Cole-Cole plots by the generalized Debye model at 1.2 kOe dc field for **2**.

T **/ K**	***τ*** **/ s**	***α***	χ_S **/ cm³ mol⁻¹**	χ_T **/ cm³ mol⁻¹**
2	6.12×10^{-3}	0.1781	0.77523	0.05322
2.5	3.5×10^{-3}	0.1358	0.63941	0.04874
3	2.11×10^{-3}	0.10997	0.54207	0.04471
3.5	1.24×10^{-3}	0.09099	0.47076	0.04111
4	7.01×10^{-4}	0.0695	0.41509	0.03894
4.5	3.82×10^{-4}	0.06026	0.37362	0.03468
5	2.00×10^{-4}	0.04129	0.33636	0.0302
5.5	1.03×10^{-4}	0.02882	0.30718	0.02379
6	4.90×10^{-5}	0.03037	0.28342	0
